# A Thickened Coracohumeral Ligament and Superomedial Capsule Limit Internal Rotation of the Shoulder Joint: Report of Three Cases

**DOI:** 10.1155/2016/9384974

**Published:** 2016-03-30

**Authors:** Masashi Koide, Junichiro Hamada, Yoshihiro Hagiwara, Kenji Kanazawa, Kazuaki Suzuki

**Affiliations:** ^1^Department of Orthopaedic Surgery, Tohoku University Graduate School of Medicine, 1-1 Seiryo-machi, Aoba-ku, Sendai 980-8574, Japan; ^2^Department of Orthopaedic Surgery, Kuwano Kyoritsu Hospital, 2-9-18 Shima, Koriyama 963-8034, Japan; ^3^Department of Orthopaedic Surgery, Niigata University Graduate School of Medical and Dental Sciences, 757 Ichibancho, Asahimachidori, Chuo-ku, Niigata 951-8510, Japan

## Abstract

Adhesive capsulitis of the shoulder (also known as frozen shoulder) is a painful and disabling disorder with an estimated prevalence ranging from 2% to 5% in the general population. Although the precise pathogenesis of frozen shoulder is unclear, thickened capsule and coracohumeral ligament (CHL) have been documented to be one of the most specific manifestations. The thickened CHL has been understood to limit external rotation of the shoulder, and restriction of internal rotation of the shoulder has been believed to be related to posterior capsular tightness. In this paper, three cases of refractory frozen shoulder treated through arthroscopic release of a contracted capsule including CHL were reported. Two cases in which there is recalcitrant severe restriction of internal rotation after manipulation under anesthesia (MUA) were finally treated with arthroscopic surgery. Although MUA could release the posterior capsule, internal rotation did not improve in our cases. After release of the thickened CHL, range of motion of internal rotation was significantly improved. This report demonstrates the role of the thickened CHL in limiting the internal rotation of the shoulder. We highlight the importance of release of thickened CHL in addition to the pancapsular release, in case of severe limitation of internal rotation of shoulder.

## 1. Introduction

Although the precise parthenogenesis of adhesive capsulitis of the shoulder (frozen shoulder) is unclear, fibrotic, inflammatory, and chondrogenic processes of the joint capsule are described as potential pathomechanisms [1, 2]. Surgical interventions, such as arthroscopic capsular release, have provided good results; therefore, joint capsulitis with both thickening and tightening has been considered to be one of the main pathologies of the disorder.

A thickened coracohumeral ligament (CHL) has been documented as one of the most specific manifestations of frozen shoulder [3, 4]. Histological analysis has detected fibrosis in the thickened CHL in frozen shoulders [3, 5]. The CHL has been described as originating from the base and the horizontal limb of the coracoid process, enclosing the subscapularis tendon, the supraspinatus tendon, and the infraspinatus tendon [6]. Although a thickened CHL that covers the rotator interval has been understood to limit shoulder joint external rotation [7], thickening of the ligament, especially from the base of the coracoid process to the superomedial capsule, would also restrict internal rotation such as hand behind back (HBB), horizontal flexion, and internal rotation in flexion or abduction. However internal rotation in a neutral position is unaffected in patients with frozen shoulder. The fact that restriction of internal rotation in a neutral position is absent in patients with frozen shoulder implies no presence of posterior capsular tightness. Hence, other soft tissue stiffness would be related to the limitation of internal rotation in nonneutral positions. The aim of this report was to demonstrate that a thickened CHL and superomedial capsule can be a cause of restricting internal rotation of the shoulder in three patients with refractory frozen shoulder.

## 2. Case Presentation

**Case 1 .**

A 57-year-old Japanese male with diabetes mellitus visited our hospital complaining that he had been suffering from bilateral shoulder pain for several months. A physical examination demonstrated that he had severe restrictions in the range of motion (ROM) in both shoulders. ROM results for the left shoulder are shown in Table 1. Preoperative magnetic resonance imaging of the left shoulder revealed a contracted inferior glenohumeral ligament (IGHL) in the axillary pouch and a thickened CHL. He was diagnosed with bilateral frozen shoulder and received physical therapy for three months; however the restriction in the ROM did not improve through physical therapy. The patient was scheduled for arthroscopic capsular release of the left shoulder. Prior to surgery the patient received a manipulation under general anesthesia to remove the restriction of the internal rotation (HBB, horizontal flexion, and internal rotations in flexion and abduction).

**Figure 1:**
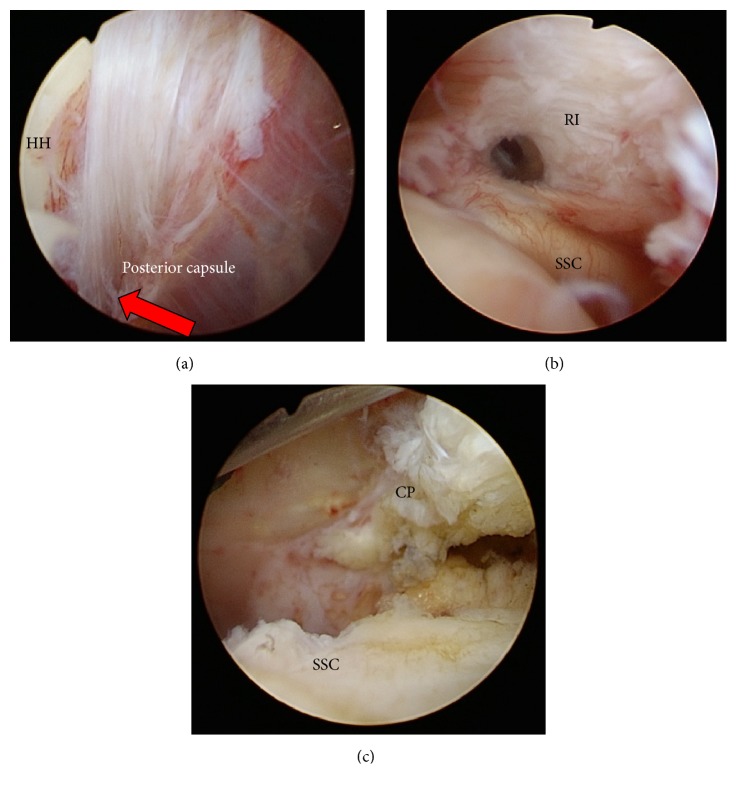
Arthroscopic findings in Case 1. SSC: subscapularis; RI: rotator interval; HH: humeral head; CP: coracoid process; PIGHL: posterior band of inferior glenoid humeral ligament. (a) Arrow shows that the posterior capsule was cut with a fresh stump. (b) Thickened RI and CHL remained. (c) Thickened anterior side of the CHL can be seen from the joint side.

Arthroscopic surgery after the manipulation showed the presence of synovial proliferation around the rotator interval, CHL, middle glenohumeral ligament, and the anterior band of the inferior glenohumeral ligament. The posterior IGHL (PIGHL) and posterior capsule had been ruptured with a fresh stump (Figure 1(a)); however, the thickened CHL remained (Figure 1(b)). After release of the rotator interval, the thickened CHL that covered both anteriorly and posteriorly the subscapularis tendon still remained. The thickened anterior side of the CHL was resected to visualize the coracoid base (Figure 1(c)), and a portion of the CHL between the subscapularis tendon and the labrum was also resected. After the complete resection of the thickened CHL, a smooth sliding movement of the subscapularis with internal and external rotation of the shoulder joint could be achieved. The sliding motion of the supraspinatus was also inhibited due to adhesion in the superomedial capsule because of synovial proliferation. The release of the adhesion between the superomedial capsule and the long head of the biceps (LHB) allowed the supraspinatus to slide smoothly. Additionally, the nonruptured anterior and inferior capsules were released by manipulation. Three weeks after the operation, the patient had almost regained full ROM in the shoulder joint and specifically internal rotation. Internal rotation ROM was fully regained and the patient reported no pain or inability to perform activities of daily life or sports activities 8.6 months after the operation (Table 1).

In this case, the posterior capsule and PIGHL were ruptured with manipulation under anesthesia (MUA) by applying a controlled force to the humerus toward internal rotation such as HBB, horizontal flexion, and internal rotation in flexion or abduction.

**Case 2 .**

**Figure 2:**
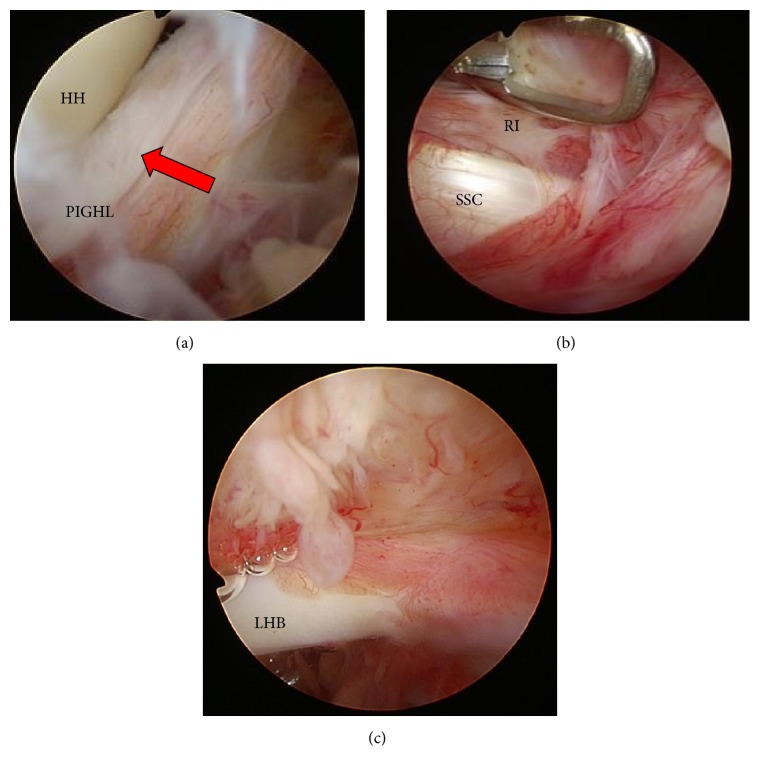
Arthroscopic findings in Case 2. LHB: long head of the biceps tendon. (a) Posterior capsule was partially ruptured by manipulation under anesthesia. (b) Before release of the rotator interval. (c) Proliferation of bursa and adhesion of the superior capsule.

A 53-year-old Japanese male with no general medical history or trauma visited our hospital due to pain in the left shoulder that had persisted for several years. A physical examination revealed a limited ROM in the left shoulder (Table 1). X-rays revealed no abnormality and he was diagnosed with refractory frozen shoulder. He received MUA 2 weeks after his first examination and then received physical therapy for 6 months. Despite improvements in flexion, abduction, and external rotation, the treatment results for internal rotation, such as HBB, and internal rotation in flexion and abduction were unacceptable to him due to occupational demands. The patient finally asked to undergo arthroscopic capsular release (Table 1). Arthroscopic surgery showed that the PIGHL and posterior capsule had been ruptured by MUA (Figure 2(a)) but that the thickened CHL in the rotator interval and the proliferation of the anterior and superior capsules remained (Figures 2(b) and 2(c)). A resection of the thickened CHL and a thickened superomedial capsular release were performed. After resection of the CHL and the capsular release, the ROM was completely regained, most noticeably in internal rotation (Table 1).

This case showed firstly that the initial MUA could rupture the posterior capsule and PIGHL, as in Case 1, but that a restriction of internal rotation could still remain. Secondly, arthroscopic resection of the thickened CHL and superomedial capsule release could recover internal rotation ROM to similar levels as that of the unaffected shoulder.

**Case 3 .**

**Figure 3:**
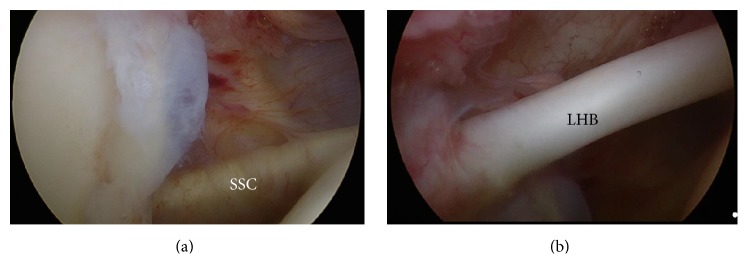
Arthroscopic findings in Case 3. (a) Synovial proliferation in the rotator interval and the superomedial capsule. (b) The CHL from the coracoid process to the LHB was thickened and inflamed.

A 53-year-old Japanese male with no past medical history visited our hospital with discomfort in the right shoulder. A physical examination indicated that there was moderate limitation in the ROM (Table 1), and the patient was diagnosed with frozen shoulder. The patient received physical therapy for 2 months and while ROM was restored in flexion and external rotation, the internal rotation ROM was not restored. The patient was unsatisfied with these results and requested arthroscopic capsular release. The arthroscopic findings revealed slight synovial proliferation in the rotator interval and the superomedial capsule and that the CHL from the coracoid process to the LHB was thickened and inflamed (Figures 3(a) and 3(b)). A resection of the thickened CHL, coupled with superomedial capsular release, was performed in the same manner as in the previous cases. 3.3 months after the operation, all ROMs, including internal rotation, were regained and the patient could perform daily life activities and job without pain (Table 1).

Even though physical therapy improved the angles of flexion, abduction, and external rotation, the remaining limitation of internal rotation was removed and ROM was restored through arthroscopic resection of the thickened CHL and the superomedial capsule.

## 3. Discussion

The most important finding of this study was the significant improvement in internal rotation after arthroscopic resection of the thickened CHL and the superomedial capsule; therefore, a thickened CHL and the superomedial capsule could conceivably be related to internal rotation limitation. Additionally, the rupture of the posterior capsule as caused by MUA did not contribute to the restoration of internal rotation in patients with frozen shoulder. A thickened CHL has been considered to be one of the most characteristic manifestations of frozen shoulder [3]. However, numerous authors have indicated that the CHL is the primary restraint against external rotation in frozen shoulder [7]. Neer et al. reported an increase of 32 degrees of external rotation after a release of the CHL in anatomic specimens of the shoulder [8], and Ozaki et al. reported that a fibrotic and thickened CHL leads to a restriction of external rotation [5]. The thickening of the CHL in the rotator interval has been considered to be related to external rotation, but the thickened CHL from the coracoid base to the superomedial capsule is responsible for the restriction in internal rotation.

Anatomic knowledge of the CHL in literature has been accumulating to develop a functional understanding of the CHL. Recent studies suggest that the CHL envelops vaster areas than had been expected. Macroscopically, the CHL was divided into 2 parts: one part spread fibers over the rotator interval to the posterior portion of the greater tuberosity and the other part enveloped the superior portion of the subscapularis, supraspinatus, and infraspinatus tendons. The anterior CHL holds the subscapularis muscle and anchors the muscle to the coracoid process in a similar manner to that of the posterior CHL enveloping the supraspinatus and infraspinatus [9, 10]. Histological analysis has demonstrated that the external rotation limitation is due to the fibroblastic proliferation of the CHL within the rotator interval and results in the loss of excursion between the subscapularis and supraspinatus tendons.

Restriction of internal rotation of the shoulder joint has been believed to be related to posterior capsular tightness [11, 12]. Burkhart et al. reported a contracted and thickened posteroinferior recess and capsule as typical arthroscopic findings in patients with limited internal rotation [11]. Tehranzadeh et al. reported posterior capsular fibrosis in professional baseball pitchers with glenohumeral internal rotation deficiencies [12]. Although excess movement of internal rotation by MUA could release the posterior capsule, internal rotation did not improve in our cases, which means that the limitation in internal rotation could be accounted for by a thickened CHL from the base of the coracoid process to superomedial capsule. The finding can explain why the restriction of internal rotation in the neutral position is absent in patients with frozen shoulder, even though restriction of other internal rotations such as HBB, horizontal flexion, and internal rotation in flexion and abduction are present. To completely understand the pathology of thickening of the CHL leading to a limitation in internal rotation of the shoulder, further study such as anatomic, biomechanical, or clinical research is necessary in the future.

## Supplementary Material

The supplementary video file summarized the arthroscopic surgery of case 1. The technique of the release of the adhered CHL and superomedial capsule are demonstrated.

## Figures and Tables

**Table 1:** Range of motion and visual analogue scale of Cases 1, 2, and 3.

	AE	LE	1st ER	HBB	HF	2nd ER	2nd IR	3rd ER	3rd IR	VAS
Case 1										
The initial visit	70	70	15	**butt**	**15**	*30*	***−20***	*30*	***−20***	7.7
Rehabilitation for 2 months	90	80	10	**butt**	**10**	*30*	***−20***	*30*	***−20***	6.4
After op for 8.6 months	165	160	65	**Th6**	**60**	90	**90**	95	**45**	0.6
Case 2										
The initial visit	110	75	10	**butt**	**10**	*10*	***20***	*30*	***0***	6.4
After MUA for 2 months	155	140	50	**L4**	**50**	85	**30**	95	**0**	5.0
After op for 4.0 months	170	180	60	**Th7**	**70**	100	**90**	105	**40**	0
Case 3										
The initial visit	110	90	45	**L2**	**30**	70	**30**	80	**15**	4.4
After rehabilitation for 6 months	150	110	70	**L4**	**30**	90	**30**	100	**20**	2.4
After op for 3.3 months	180	180	70	**Th7**	**60**	90	**90**	110	**70**	0

Internal rotation (bold font) is significantly improved after arthroscopic capsular release. The 2nd ER and IR and the 3rd ER and IR (italic font) were measured at the maximum range of motion of elevation.

AE: anterior elevation; LE: lateral elevation; 1st ER: external rotation in adduction of the shoulder joint; HBB: the highest posterior spinal level reached when hand is behind back; HF: horizontal flexion; 2nd ER: external rotation in 90 degrees of lateral elevation; 2nd IR: internal rotation in 90 degrees of anterior elevation; 3rd ER: external rotation in 90 degrees of anterior flexion; 3rd IR: internal rotation in 90 degrees of anterior elevation; VAS: visual analogue scale.
